# Accidental exposure (AE) to peanut in a large cohort of Canadian children with peanut allergy

**DOI:** 10.1186/1710-1492-10-S1-A32

**Published:** 2014-03-03

**Authors:** Sabrine Cherkaoui, Moshe Ben-Shoshan, Reza Alizadehfar, Yuka Asai, Greg Shand, Yvan St-Pierre, Laurie Harada, Mary Allen, Ann Clarke

**Affiliations:** 1 Division of Internal Medicine, Department of Medicine, University of Montreal, Montreal, QC, Canada; 2 Division of Pediatric Allergy and Clinical Immunology, Department of Pediatrics, McGill University Health Center, Montreal, QC, Canada; 3 Division of Dermatology, Department of Medicine, McGill University Health Center, Montreal, QC, Canada; 4 Division of Clinical Epidemiology, Department of Medicine, McGill University Health Center, Montreal, QC, Canada; 5 Anaphylaxis Canada (AC), Toronto, ON, Canada; 6 Allergy/Asthma Information Association (AAIA), Toronto, ON, Canada; 7 Division of Clinical Immunology and Allergy, Department of Medicine, McGill University Health Center, Montreal, QC, Canada

## Background

We have previously estimated that the annual rate of accidental exposure (AE) to peanut in a Canadian cohort of 1411 children with peanut allergy, followed for 2227 patient-years, was 11.9% [1]. The cohort has increased to 1825 children, with 4134 patient-years of follow-up, and we determined the incidence of AE in this expanded cohort and described the severity, management, and location of the AE.

## Methods

Children with physician-confirmed peanut allergy were identified from the Montreal Children’s Hospital and food allergy advocacy organizations from 2004 to May 2013. Parents completed a questionnaire at study entry and every two years regarding their child’s AE to peanut over the preceding year; starting in 2010, follow-up questionnaires were administered annually.

## Result

The mean age (SD) was 2.4 (2.1) years at diagnosis and 7.0 (4.0) years at the time of the initial questionnaire completion. Patients were predominantly boys (61.8%) and Caucasians (89.5%). When all children were included, regardless of length of observation interval, 456 AE occurred in 336 children over 4134 patient-years, yielding an annual incidence rate of 11.0 % (95 % CI, 9.0 - 13.1%). Because the rate of AE may vary with observation interval length, the rate was calculated excluding AE occurring after the initial questionnaire and excluding those providing <1 year of observation; this yielded 164 AE in 141 children over 1405 patient-years, for an annual incidence rate of 11.7% (95 % CI, 9.7 % - 13.6%). One hundred forty-seven AE were mild, 242 moderate, and 67 severe. Among 429 AE preceded by an initial reaction, 22.4 % of AE were more severe than the initial reaction. No treatment was administered for 41 (27.9 %) mild AE, 40 (16.5%) moderate and 4 (6.0%) severe. Of 309 AE that were moderate/severe, only 93 (30.1%) sought medical attention and among these, only 30.1% received epinephrine. Of the 153 moderate/severe AE treated at home, only (11.8%) received epinephrine. Thirty-nine percent of AE occurred at home, 17.3% at relatives/friends’ home, 11.4% in restaurants, 7.5% at schools/day-cares prohibiting peanut, 3.7% at schools/day-cares allowing peanuts, and 20.6% at other or unknown places.

## Conclusion

Despite increasing efforts to provide information on the management of food allergy, AE continue to occur, mainly in the child’s home but also in peanut free schools/day-cares. Most moderate/severe AE are managed inappropriately by caregivers and physicians. Consequently, more education is required on the importance of strict allergen avoidance and the need for prompt and correct management of anaphylaxis.
